# Dysphagia as the Presenting Symptom of Laryngeal Tuberculosis

**DOI:** 10.7759/cureus.14495

**Published:** 2021-04-15

**Authors:** Emad Kandah, Raghunandan Konda, Bilal Malik, Adan Madadha, Arvind Kunadi

**Affiliations:** 1 Internal Medicine, McLaren Health Care, Flint/Michigan State University (MSU), Flint, USA; 2 Internal Medicine, McLaren Flint/Michigan State University (MSU), Flint, USA; 3 Diagnostic Medical Laboratories, Cell Therapy Center/University of Jordan, Amman, JOR; 4 Internal Medicine/Nephrology, McLaren Health Care, Flint/Michigan State University (MSU), Michigan, USA

**Keywords:** dysphagia, tuberculosis, tb, laryngeal tb

## Abstract

Laryngeal tuberculosis (TB) is a rare form of tuberculosis that most commonly presents with dysphagia and weight loss. We report a case of a 75-year-old Vietnamese male who presented with dysphagia, odynophagia, and a 30-pound weight loss over the two months prior to presentation. Nasopharyngeal evaluation with microdirect laryngoscopy was performed as part of the workup and revealed lesions of the epiglottis and left vocal cord. A tissue biopsy and quantiferon testing confirmed the diagnosis of tuberculosis. The patient was started on quadruple therapy and is currently receiving treatment. This case highlights the need for consideration of the rare, yet important, differential of laryngeal TB in patients presenting with non-specific complaints such as dysphagia and weight loss.

## Introduction

Tuberculosis (TB) is a contagious disease that is almost exclusively transmitted by aerosolized respiratory secretions, with mortality approaching 70% if left untreated [1]. Globally, it is estimated that 1.7 billion people are infected with mycobacterium tuberculosis [2]. TB is characterized by necrotizing granulomas that mainly affect the lungs (~85% of cases), yet any extrapulmonary site can be involved [1,3]. In the early twentieth century, laryngeal TB accounted for 35%-83% of tuberculosis cases based on autopsy findings [4]. The incidence of laryngeal TB has dramatically decreased after the advent of anti-tubercular agents, accounting for approximately 1% of TB cases in recent times [3-5].

## Case presentation

**Figure 1:**
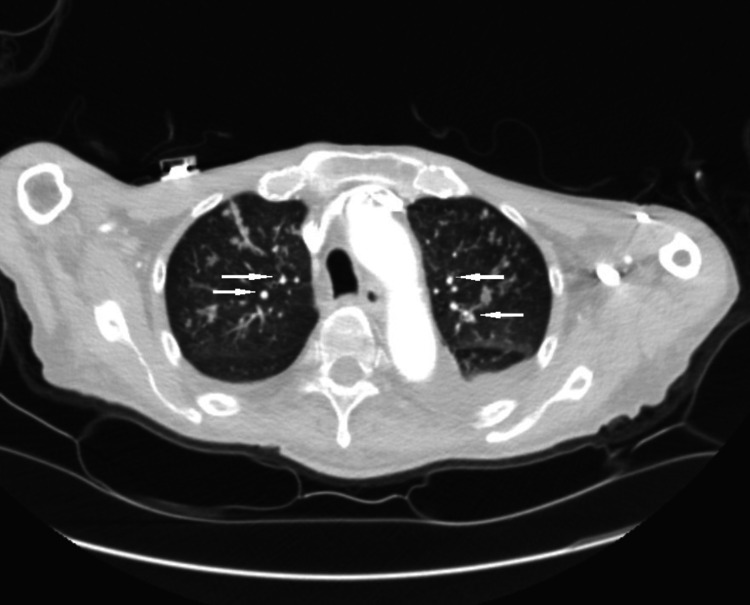
CT scan of the chest with contrast revealed bilateral, irregular nodular densities (white arrows)

**Figure 2:**
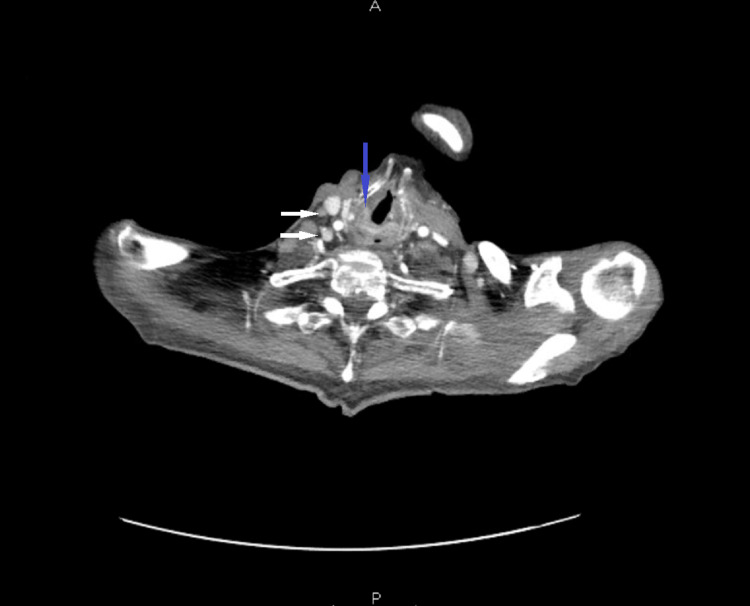
CT scan of the soft tissue of the neck with contrast showing soft tissue fullness in the cervical esophagus (blue arrow) with multiple necrotic lymph nodes throughout the cervical chains (white arrows)

A 75-year-old Vietnamese male with a past medical history significant for essential hypertension, hyperlipidemia, and chronic obstructive pulmonary disease (COPD) presented to the hospital with complaints of dysphagia, odynophagia, and a 30-pound weight loss over a two-month period. The patient denied tobacco, alcohol, or illicit drug use. At the time of presentation, the patient had a blood pressure of 160/85 mmHg, heart rate of 71 beats per minute, respiratory rate of 16 breaths per minute, oxygen saturation of 96% on ambient air, and a temperature of 98.5 F. Laboratory studies were significant for leukopenia (white blood cell (WBC) count 3.79 X 10*3/uL, reference range 4.5-11.0 X 10*3/uL) and hypokalemia (potassium level 2.9 mM/L, reference range 3.5-5.1 mM/L) but were otherwise unremarkable. Radiographic evaluation with a chest X-ray was negative for any acute cardiopulmonary processes. A computed tomography (CT) scan of the chest with contrast revealed bilateral, irregular nodular densities involving both upper lobes and the right middle lobe, worrisome for pneumonia (Figure 1). A CT scan of the soft tissue of the neck with contrast revealed 1.2 x 1.3 cm soft tissue fullness in the cervical esophagus with multiple necrotic lymph nodes throughout the cervical chains (Figure 2).

**Figure 3:**
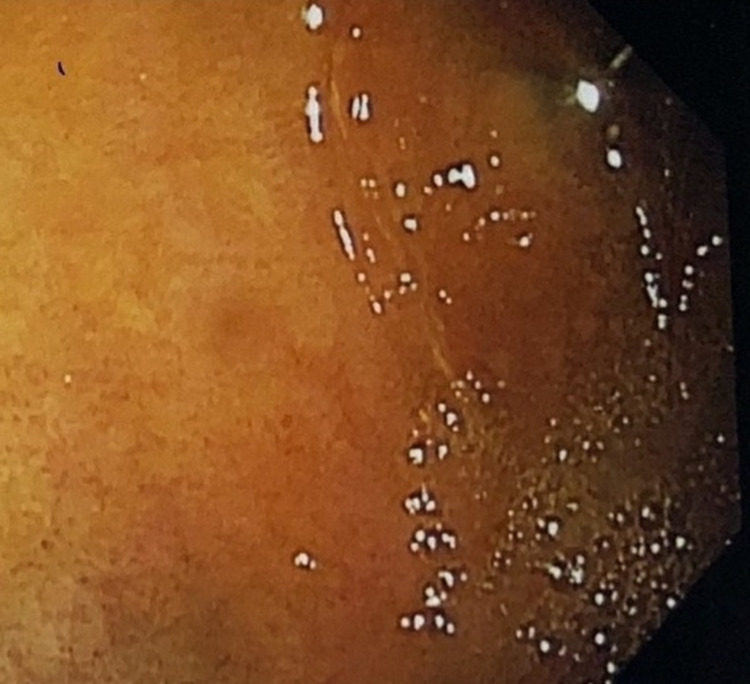
Esophagogastroduodenoscopy showing normal esophagus without any inflammation, strictures, or lesions

The patient was empirically initiated on intravenous (IV) azithromycin and ceftriaxone for the treatment of community-acquired pneumonia (CAP). Gastroenterology was consulted for further evaluation of the esophageal fullness and dysphagia. The patient underwent esophagogastroduodenoscopy (EGD), which did not reveal any inflammation, strictures, or lesions of the esophagus that aligned with the CT scan findings (Figure 3). The stomach and duodenum were unremarkable. A percutaneous endoscopic gastrostomy (PEG) tube was placed to allow the patient to receive nutrition.

**Figure 4:**
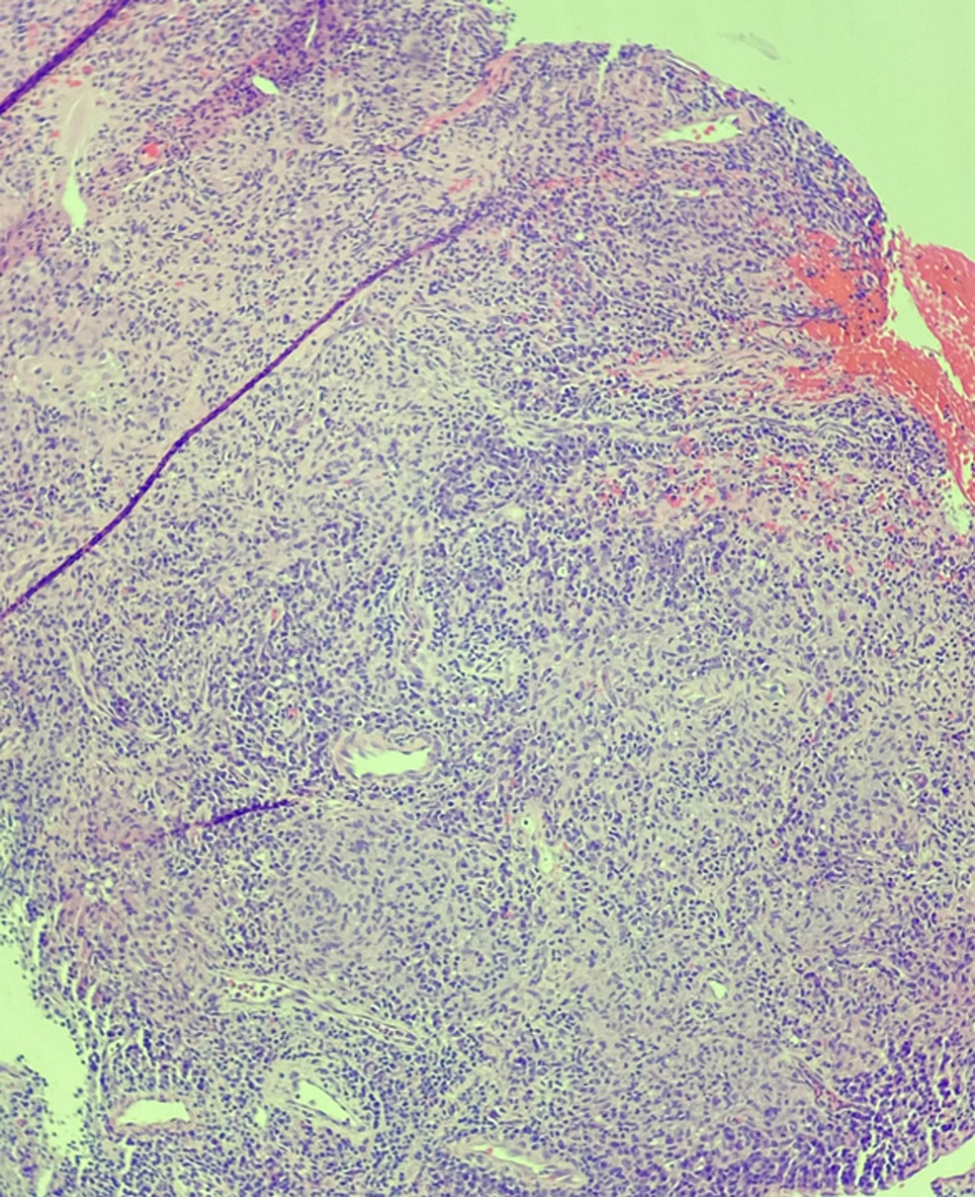
Epiglottic mucosal biopsy demonstrated fragments of squamous mucosa with extensive necrotizing granulomatous inflammation with no evidence of dysplasia or malignancy

**Figure 5:**
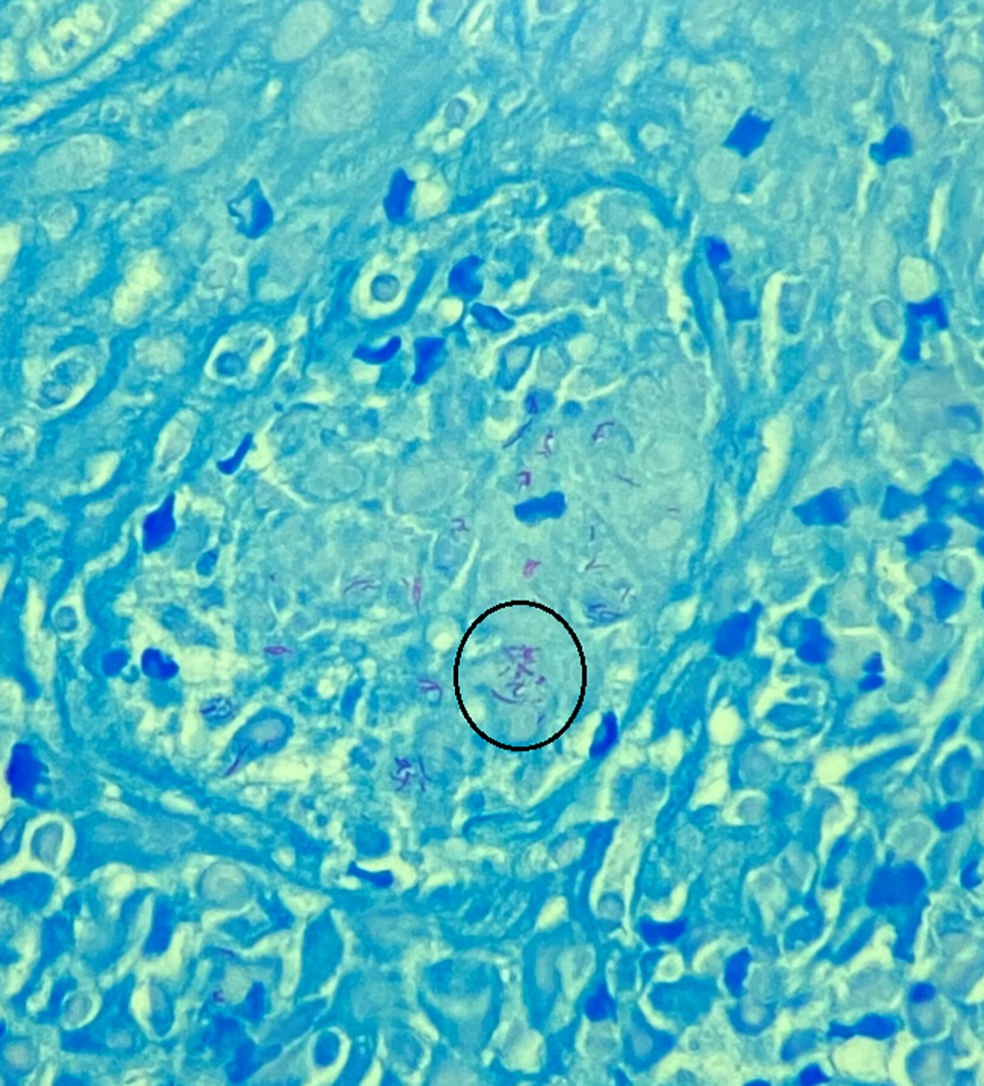
AFB special stain of the epiglottic mucosa highlights numerous acid-fast bacilli (black circle) AFB: Acid- Fast Bacilli

A nasopharyngeal evaluation with microdirect laryngoscopy was subsequently performed and revealed raised irregularity involving the laryngeal surface and lingual surface of the epiglottis. A left vocal cord nodule was also present. Multiple biopsies were obtained from the identified lesions. The left vocal cord nodule biopsy revealed a polypoid fragment of squamous mucosa with mixed inflammatory infiltrates and stromal fibrosis. There was no evidence of dysplasia or malignancy. The epiglottic mucosal biopsy demonstrated fragments of squamous mucosa with extensive necrotizing granulomatous inflammation with no evidence of dysplasia or malignancy (Figure 4). Acid-fast bacilli (AFB) stain for mycobacterial organisms returned positive (Figure 5), and Mycobacterium tuberculosis (TB) complex polymerase chain reaction (PCR) was also positive. Interferon-gamma release assay (IGRA) was performed and confirmed the diagnosis of TB. The patient was started on the standard quadruple therapy with isoniazid, rifampin, ethambutol, and pyrazinamide.

## Discussion

Tuberculosis (TB) is thought to primarily be a respiratory disease, with transmission occurring through inhalation of Mycobacterium TB [5]. We presented the case of an elderly gentleman whose ongoing complaints included dysphagia, poor appetite, and weight loss. On further evaluation, the patient was found to have a laryngeal lesion and biopsy material from these lesions demonstrated Mycobacterium species. As TB becomes less and less common in our day-to-day practices in North America, it remains an important differential in a variety of patients. Our case report illustrates the importance of maintaining a broad differential in presentations that appear routine to us in our practices.

The World Health Organization (WHO) estimated that approximately 10 million individuals became ill with newly diagnosed TB in 2019, with 1.4 million deaths (208,000 of which were human immunodeficiency virus (HIV) positive). There are 30 countries deemed “high TB burden countries” that account for 87% of the world’s cases. Of these 30 countries, India, Indonesia, China, the Philippines, Pakistan, Nigeria, Bangladesh, and South Africa account for two-thirds of total cases, in descending order. According to the same WHO report data, there has been a cumulative 9% drop in the incidence of TB worldwide between 2015 and 2019 [6]. In earlier times, laryngeal TB was a prevalent presentation, occurring in anywhere from 35%-83% of cases based on autopsy results [4]. The prevalence of laryngeal TB, representing approximately 1% of TB cases in modern times, has been declining in North America, though it is suspected that missed diagnosis and under-reporting may play a role in this trend [4,7].

Despite being a rare presentation in modern times, laryngeal TB is one of the commonest causes of granulomatous lesions of the larynx [7]. Laryngeal TB can present as a primary lesion or a secondary lesion, depending on various modes of pathogenesis [4-5,7-8]. Primary lesions occur through the direct spread of bacilli into the larynx. Secondary lesions are thought to occur via either bronchogenic spread from pulmonary TB or through hematogenous or lymphatic spread [4-5,7-8]. In older literature, the vast majority of laryngeal TB cases were found to be secondary cases in younger patients with primary pulmonary TB and a high organism load [4]. Current literature, interestingly, has shown a reversal in this trend, with the majority of cases being primary laryngeal TB in an older patient demographic [4].

Common complaints in patients with laryngeal TB include hoarseness, odynophagia, dysphagia, weight loss, and cough [4,7-9]. On presentation, many of these cases are evaluated for a malignancy workup. Many patients, like our patient, also end up undergoing upper endoscopy to further evaluate complaints of dysphagia and odynophagia. When they finally have a laryngoscopy, they are discovered to have ulcerations or growths on the vocal cords, which, when biopsied, reveal the diagnosis of TB [4-5,7-9]. Interestingly, these masses can be treated with, and respond favorably to (i.e., no surgical intervention is necessary unless stenosis is present), standard of care anti-TB therapies in areas where strains are not resistant [4-5,7-9].

## Conclusions

Today, we presented a case that highlighted a rare, but increasingly prevalent, presentation of TB in North America. Our 75-year-old Asian patient presented with ambiguous complaints of dysphagia, weight loss, and odynophagia and was later found to have laryngeal TB. Despite its overall decreasing prevalence, TB remains an important differential to maintain in our day-to-day practice as highlighted by this case report. We recommend a low threshold for focused evaluation for TB in patients with non-specific presentations with dysphagia and weight loss, especially in patients who originate from high-risk regions of the world.

## References

[1] Dheda K, Barry CE 3rd, Maartens G (2016). Tuberculosis. Lancet.

[2] Furin J, Cox H, Pai M (2019). Tuberculosis. Lancet.

[3] Wang CC, Lin CC, Wang CP, Liu SA, Jiang RS (2007). Laryngeal tuberculosis: a review of 26 cases. Otolaryngol Head Neck Surg.

[4] Benwill JL, Sarria JC (2014). Laryngeal tuberculosis in the United States of America: a forgotten disease. Scand J Infect Dis.

[5] Kiakojuri K, Hasanjani Roushan MR (2012). Laryngeal tuberculosis without pulmonary involvement. Caspian J Intern Med.

[6] (2020). WHO. Global tuberculosis report 2020. https://apps.who.int/iris/bitstream/handle/10665/336069/9789240013131-eng.pdf.

[7] Sakthivel P, Singh CA, Sharma SC, Kanodia A, Jat B, Rajeshwari M (2017). Primary laryngeal tuberculosis-“the great masquerader”. Clin Case Rep Rev.

[8] Kurokawa M, Nibu K, Ichimura K, Nishino H (2015). Laryngeal tuberculosis: a report of 17 cases. Auris Nasus Larynx.

[9] El Kettani NE, El Hassani M, Chakir N, Jiddane M (2010). Primary laryngeal tuberculosis mimicking laryngeal carcinoma: CT scan features. Indian J Radiol Imaging.

